# Enhancement of Anti-Inflammatory and Osteogenic Abilities of Mesenchymal Stem Cells via Cell-to-Cell Adhesion to Periodontal Ligament-Derived Fibroblasts

**DOI:** 10.1155/2017/3296498

**Published:** 2017-01-12

**Authors:** Keita Suzuki, Naoyuki Chosa, Shunsuke Sawada, Naoki Takizawa, Takashi Yaegashi, Akira Ishisaki

**Affiliations:** ^1^Division of Cellular Biosignal Sciences, Department of Biochemistry, Iwate Medical University, Yahaba, Iwate 028-3694, Japan; ^2^Division of Periodontology, Department of Conservative Dentistry, Iwate Medical University School of Dentistry, Morioka, Iwate 020-8505, Japan; ^3^Department of Otolaryngology, Dentistry and Oral Surgery, Kansai Medical University, Hirakata, Osaka 573-1010, Japan

## Abstract

Mesenchymal stem cells (MSCs) are involved in anti-inflammatory events and tissue repair; these functions are activated by their migration or homing to inflammatory tissues in response to various chemokines. However, the mechanism by which MSCs interact with other cell types in inflammatory tissue remains unclear. We investigated the role of periodontal ligament fibroblasts (PDL-Fs) in regulating the anti-inflammatory and osteogenic abilities of bone marrow-derived- (BM-) MSCs. The expression of monocyte chemotactic protein- (MCP-)1 was significantly enhanced by stimulation of PDL-Fs with inflammatory cytokines. MCP-1 induced the migratory ability of BM-MSCs but not PDL-Fs. Expression levels of anti-inflammatory and inflammatory cytokines were increased and decreased, respectively, by direct-contact coculture between MSCs and PDL-Fs. In addition, the direct-contact coculture enhanced the expression of MSC markers that play important roles in the self-renewal and maintenance of multipotency of MSCs, which in turn induced the osteogenic ability of the cells. These results suggest that MCP-1 induces the migration and homing of BM-MSCs into the PDL inflammatory tissue. The subsequent adherence of MSCs to PDL-Fs plays an immunomodulatory role to terminate inflammation during wound healing and upregulates the expression stem cell markers to enhance the stemness of MSCs, thereby facilitating bone formation in damaged PDL tissue.

## 1. Introduction

Mesenchymal stem cells (MSCs) are adult stem cells with the ability to differentiate into mesenchymal cells such as osteoblasts, adipocytes, chondrocytes, and fibroblasts, while retaining self-renewal and migration abilities [1]. MSCs were initially identified in the bone marrow by Friedenstein et al. [2, 3]. Subsequently, MSCs were isolated from the adipose tissues [4], fetal liver [5], cord blood and mobilized peripheral blood [6], fetal lung [7], placenta [8], umbilical cord [9, 10], dental pulp [11], synovial membrane [12], periodontal ligament (PDL) [13], endometrium [14], and trabecular and compact bone [15, 16].

Upon activation by tissue damage in vivo, MSCs contribute to tissue repair through a multitude of processes such as self-renewal, migration, and differentiation. Cell migration is closely related to stem cell homing. Stem cell therapy relies on the appropriate homing and engraftment capacity of stem cells. Chemokines such as monocyte chemotactic protein-1 (MCP-1/CCL2) and/or stromal cell-derived factor-1 (SDF-1/CXCL12) and their receptors such as CCR2 and CXCR4 promote the effective homing of MSCs. The CXCR4 ligand SDF-1 has a dose-dependent effect on human and murine bone marrow-derived MSC (BM-MSC) migration [17–19]. Kanbe et al. [20] demonstrated that synovial fibroblasts secrete high levels of SDF-1 in osteoarthritis and rheumatoid arthritis. This raises the possibility that the SDF-1 secreted in arthritic joints, and its action as an MSC chemoattractant, directs MSC homing. In addition, our previous study suggested that SDF-1 secreted from dental pulp and PDL cells retains the ability to promote the recruitment of BM-MSCs [21–23]. MCP-1 is a chemokine that is induced under conditions of oxidative stress [24]. Recently, we proposed a novel mechanism for the promotion of the migration of BM-MSCs via the scrapie responsive gene 1 (SCRG1)/bone marrow stromal cell antigen 1 (BST1) axis through the activation of the FAK/PI3K/Akt signaling pathway in an autocrine/paracrine manner [25]. Our results also suggested that the SCRG1/BST1 axis promotes the tissue-regenerative ability of MSCs by stimulating and maintaining their stem cell activity.

Many recent studies have demonstrated that MSCs possess immunomodulatory properties [26, 27]. The immunosuppressive effect of transplanted MSCs has also been demonstrated in acute severe graft-versus-host disease [28] and in multiple-system atrophy [29]. In addition, MSCs can induce peripheral tolerance and migrate to injured tissues, where they can inhibit the release of proinflammatory cytokines and promote the survival of damaged cells [26]. For example, the therapeutic benefit of MSC transplantation has been observed in acute lung injury [30], myocardial infarction [31], acute renal failure [32], cerebral ischemia [33], and Alzheimer's disease [34]. MSCs can directly inhibit the proliferation of T lymphocytes and microglial cells and can negatively modulate the cytokine-secretion profile of dendritic cells and monocytes and/or macrophages [35–38].

Previously, we reported that the expression levels of inflammation-related chemokines associated with MCP-1 were enhanced by stimulation with IL-1*β* and/or IL-6/sIL-6R in gingival fibroblasts [39]. The aim of the present study was to investigate the regulatory mechanism of PDL-fibroblasts (PDL-Fs) on the anti-inflammatory and osteogenic abilities of BM-MSCs. We examined the expression of MCP-1 in PDL-Fs stimulated with the inflammatory cytokines interleukin (IL)-1*β*, IL-6/soluble IL-6 receptor (sIL-6R), or tumor necrosis factor (TNF)-*α*, and its effect on the recruitment of MSCs into PDL inflammatory tissues in vivo. Furthermore, we used a direct-contact coculture system between MSCs and PDL-Fs to examine the expression of anti-inflammatory and inflammatory cytokines and stem cell markers known to play an important role in the self-renewal and maintenance of multipotency of MSCs. These results can provide insight into the molecular mechanism and key factors contributing to the migration and homing of BM-MSCs into the PDL inflammatory tissue, which can be useful for enhancing bone regeneration in damaged PDL tissue with stem cell therapy.

## 2. Materials and Methods

### 2.1. Cytokines

Recombinant human IL-1*β*, IL-6, TNF-*α*, MCP-1, and SDF-1*α* were purchased from Miltenyi Biotec (Bergisch Gladbach, Germany). The cells were treated with 10 ng/mL of IL-1*β*, IL-6, TNF-*α*, MCP-1, and SDF-1*α* at various time points. Soluble IL-6 receptor (sIL-6R) was provided by Prospec-Tany TechnoGene (Ness Ziona, Israel). IL-6 was added in conjunction with 10 ng/mL of sIL-6R [39].

### 2.2. Cell Culture

We previously reported the process for the establishment and culture method for MSC lines derived from the bone marrow of mice expressing green fluorescent protein (GFP) [40, 41]. SG2 cells, a transforming growth factor (TGF)-*β*-responsive MSC line, were cultured in Dulbecco's modified Eagle's medium (DMEM; Sigma-Aldrich, St. Louis, MO, USA) supplemented with 10% fetal bovine serum (FBS; BI Biological Industries, Kibbutz Beit Haemek, Israel) at 37°C under hypoxic conditions (5% O_2_, 5% CO_2_, and 90% N_2_). The isolation of rat PDL-Fs and establishment of single cell-derived cultures (SCDCs) have been previously described [42]. SCDC2 cells were cultured on type I collagen-coated plastic dishes (Sumilon Celltight Plate, Sumitomo Bakelite Co., Tokyo, Japan) in Ham's F-12 (Sigma-Aldrich) supplemented with 2 mM glutamine (100x solution; Gibco), 10% FBS, 10 ng/mL fibroblast growth factor (FGF)-1 (R&D Systems Inc., Minneapolis, MN, USA), 15 *μ*g/mL heparin (Sigma-Aldrich), and penicillin (Gibco, Carlsbad, CA, USA) in a humidified atmosphere of 5% CO_2_ at 37°C. Human gingival fibroblasts (HGFs) were cultured in DMEM (Sigma-Aldrich) supplemented with 10% FBS (BI Biological Industries) at 37°C under 5% CO_2_ according to our previous report [39].

### 2.3. Coculture System

SG2 cells and SCDC2 cells or HGFs were cultured in a direct coculture system and indirect coculture system. Direct coculture (CC) was performed in a monolayer at a 50 : 50 ratio (4.0 × 10^5^ cell/well) in six-well plates (Sumilon) [43]. Indirect coculture was performed in a transwell coculture system (TW) [44]. The TW coculture system consisted of a polycarbonate transwell chamber, which can be inserted into the well of standard 24-well plates. SG2 cells (1.0 × 10^5^ cell/well) were seeded on the bottom of the 24-well culture plates. Then, SCDC2 cells or HGFs (1.0 × 10^5^ cell/well) were seeded on the upper membrane (pore size of 0.4 *μ*m) of the transwell chamber. The cells were maintained in Ham's F-12 supplemented with 2 mM glutamine (100x solution), 10% FBS, 10 ng/mL FGF-1, 15 *μ*g/mL heparin, and penicillin in a humidified atmosphere of 5% CO_2_ at 37°C.

### 2.4. Quantitative Reverse Transcription-Polymerase Chain Reaction (RT-qPCR)

Total RNAs were isolated with ISOGEN reagent (Nippon Gene, Toyama, Japan) and first-stand cDNA was synthesized with the PrimerScript RT Reagent Kit (Takara Bio, Shiga, Japan) according to the manufacturer's instructions. RT-qPCR was performed on a Thermal Cycler Dice Real Time System (Takara Bio) with SYBR Premix Ex Taq II (Takara Bio) and specific oligonucleotide primers (Table 1) using a two-step cycle procedure (denaturation at 95°C for 5 s and annealing and extension at 60°C for 30 s) for 40 cycles. For each PCR run, cDNA derived from 50 ng total RNA as a template and 0.4 *μ*M of each primer pair was used. The mRNA expression level was normalized to that of glyceraldehyde 3-phosphate dehydrogenase* (Gapdh)*, and the relative amount of each mRNA in each sample was calculated using the ΔΔCq method. The relative mRNA expression levels are expressed as the fold increase or decrease relative to the control.

### 2.5. Enzyme-Linked Immunosorbent Assay (ELISA)

SCDC2 cells were stimulated with or without 10 ng/mL of IL-1*β*, IL-6, or TNF-*α* for 48 h. The amount of secreted chemokines was measured using sandwich ELISA kits for rat MCP-1 (R&D Systems Inc.) and rat SDF-1*α* (Abnova, Taipei City, Taiwan). SG2 cells were directly cocultured with SCDC2 cells for 48 h. The cytokines produced from the SG2 cells into the medium were quantified using sandwich ELISA kits for mouse IL-10 (R&D Systems Inc.), mouse TGF-*β* (Abcam, Cambridge, UK), and mouse IL-6 (R&D Systems Inc.) with specific antibodies that do not cross-react with rat IL-10, TGF-*β*, and IL-6. The target proteins were measured according to the manufacturer's instructions. The absorbance was measured using an MPR-A4i microplate reader (Tosoh Corp., Tokyo, Japan).

### 2.6. Flow Cytometry Analysis

SG2 cells directly or indirectly cocultured with SCDC2 cells were suspended in ice-cold phosphate-buffered saline (PBS) containing 0.5% FBS and 2 mM ethylenediaminetetraacetic acid (EDTA). The cells were incubated with phycoerythrin- (PE-) conjugated anti-mouse SCA-1, CD44, and CD90 antibodies (Miltenyi Biotec) for 1 h at 4°C in the dark. Acquisition was performed with an EPICS XL ADC System (Beckman Coulter, Brea, CA, USA). Measurement of fluorescence intensity relative to PE in SG2 cells was performed after gating according to the fluorescence of GFP.

### 2.7. Transwell Migration Assay

The migration assay was performed as described previously [25], using transwell cell culture inserts (BD Bioscience, Franklin Lakes, NJ, USA) that were 6.5 mm in diameter with 8 *μ*m pore filters. The cells (5.0 × 10^4^ cells/well) were suspended in 350 *μ*L of serum-free DMEM containing 0.1% bovine serum albumin (Sigma-Aldrich) and seeded into the upper well; 600 *μ*L of 10% FBS-supplemented DMEM with or without 10 ng/mL MCP-1 and 100 ng/mL anti-MCP-1 neutralizing antibody (Abcam) was placed in the lower well of the transwell plate. After incubation for 6 h at 37°C under hypoxic conditions, cells that had not migrated from the upper side of the filter were scraped off with a cotton swab, and the membrane was fixed in 4% paraformaldehyde in PBS. After washing with PBS, the cells that had migrated onto the underside of the membrane were labeled with DAPI (1 : 1,000; Kirkegaard & Perry Laboratories, Gaithersburg, MD, USA) and counted under a fluorescence microscope in five high-power fields (400x magnification; Olympus IX70; Olympus Corp., Tokyo, Japan).

### 2.8. Stemness Investigation

SCDC2 cells as feeder cells were precultured to confluence and then fixed with methanol. SG2 cells were CC cocultured on the fixed feeder SCDC2 cells.

Osteogenic and adipogenic differentiation was reported in our previous paper [25]. The CC cocultured SG2 cells were incubated in osteogenic induction medium for 10 days. Bone matrix mineralization was evaluated by alizarin red S (Sigma) staining. Alizarin red S was extracted by adding 10% cetylpyridinium chloride (Sigma) in 8 mM Na_2_HPO_4_ (Merck Millipore, Darmstadt, Germany) and 1.5 mM KH_2_PO_4_ (Merck), and absorbance was measured at 540 nm using an MPR-A4i microplate reader (Tosoh Corp.). To induce adipogenic differentiation, CC cocultured SG2 cells were cultured in adipogenic differentiation medium for 5 days. Lipid droplets were stained with Oil Red O (Sigma-Aldrich). Oil Red O stain was quantified by extraction from lipid droplets with dimethyl sulfoxide (DMSO, Sigma) and absorbance was measured at 540 nm.

Cell proliferation was analyzed using a colorimetric assay for cleavage of the tetrazolium salt WST-1 (Roche Diagnostics, Basel, Switzerland) by mitochondrial dehydrogenases in viable cells. The CC cocultured SG2 cells were cultured in growth medium for 6 days. Cell proliferation was evaluated by measuring the absorbance at 450 nm on an MPR-A4i microplate reader (Tosoh Corp.) according to the manufacturer's instructions.

SG2 cells CC cocultured for 48 h were stripped by 0.25% trypsin containing 1 mM EDTA and the migratory ability was investigated as described previously.

### 2.9. Statistical Analysis

All experiments were repeated at least three times. Representative images or data are shown. The numerical data are presented as the mean ± standard deviation. Differences in the mean and percentages between the control and treatment groups were statistically analyzed using paired two-tailed Student's *t*-tests, and *P* < 0.05 was considered statistically significant.

## 3. Results

### 3.1. Inflammatory Cytokines Promote MCP-1 Production in SCDC2 Cells

**Figure 1:**
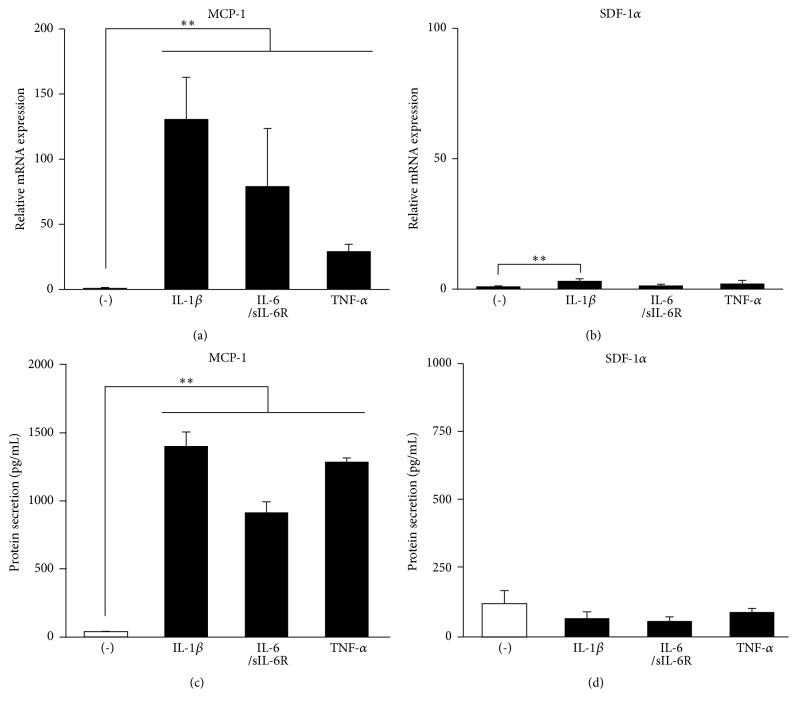
Inflammatory cytokines promote MCP-1 production in SCDC2 cells. SCDC2 cells were stimulated with or without 10 ng/mL of IL-1*β*, IL-6/sIL-6R, or TNF-*α*. (a, b) mRNA expression levels were investigated by RT-qPCR using rat-specific primers. Reported values are normalized to* Gapdh* expression. The results are expressed as the fold change relative to the respective control (-). (c, d) The amount of secreted chemokines was measured using sandwich ELISA kits for rat-specific MCP-1 and SDF-1*α*. Data are presented as the mean ± standard deviation. ^*∗*^*P* < 0.05 and ^*∗∗*^*P* < 0.01 as compared with the unstimulated control (-).

As shown in Figure 1, MCP-1 mRNA expression and protein secretion into the culture medium were significantly enhanced by stimulation with inflammatory cytokines such as IL-1*β*, IL-6, and TNF-*α* in the PDL-F SCDC2 cells (Figures 1(a) and 1(b)). By contrast, the secretion of SDF-1*α* protein, which is a chemokine involved in the migration of MSCs, similar to MCP-1, was not enhanced by stimulation with these cytokines in SCDC2 cells (Figures 1(c) and 1(d)). These results indicated that inflammatory cytokines specifically enhanced the production of MCP-1 in PDL-Fs.

### 3.2. MCP-1 Specifically Induces the Migration of SG2 Cells Rather Than That of SCDC2 Cells

**Figure 2:**
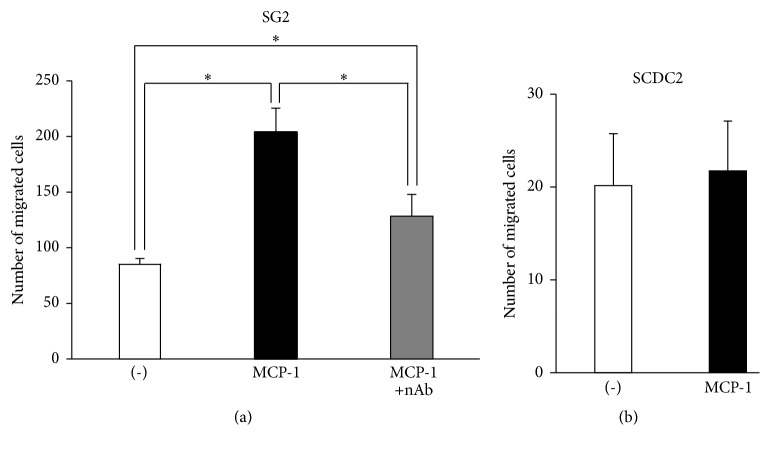
MCP-1 specifically induces the migration of SG2 cells rather than that of SCDC2 cells. Transwell migration assay for SG-2 cells (a) and SCDC2 cells (b) by stimulation with 10 ng/mL of MCP-1. Anti-MCP-1 neutralizing antibody (nAb) was also added with MCP-1 for migration in SG2 cells (a). The cells that migrated to the underside of the membrane were labeled with DAPI and counted under a fluorescence microscope in five high-power fields. Data are presented as the mean ± standard deviation. ^*∗*^*P* < 0.05 as compared with the unstimulated control (-) for each cell line.

MCP-1 is a chemokine that is known to strongly chemoattract MSCs through interaction with its unique receptor, CCR2. We therefore investigated the migratory activity of the mouse BM-MSC line SG2 stimulated by MCP-1. The number of transwell-migrated SG2 cells stimulated with MCP-1 was more than double that of the control cells (Figure 2(a)); this induction was completely canceled by anti-MCP-1 neutralizing antibody. In contrast, MCP-1 did not induce the migratory activity of SCDC2 cells (Figure 2(b)). These results indicated that MCP-1 specifically induced the migratory activity of MSCs rather than that of PDL-Fs.

### 3.3. Expression of Anti-Inflammatory Cytokines in SG2 Cells Is Increased by Direct Coculture with SCDC2 Cells

**Figure 3:**
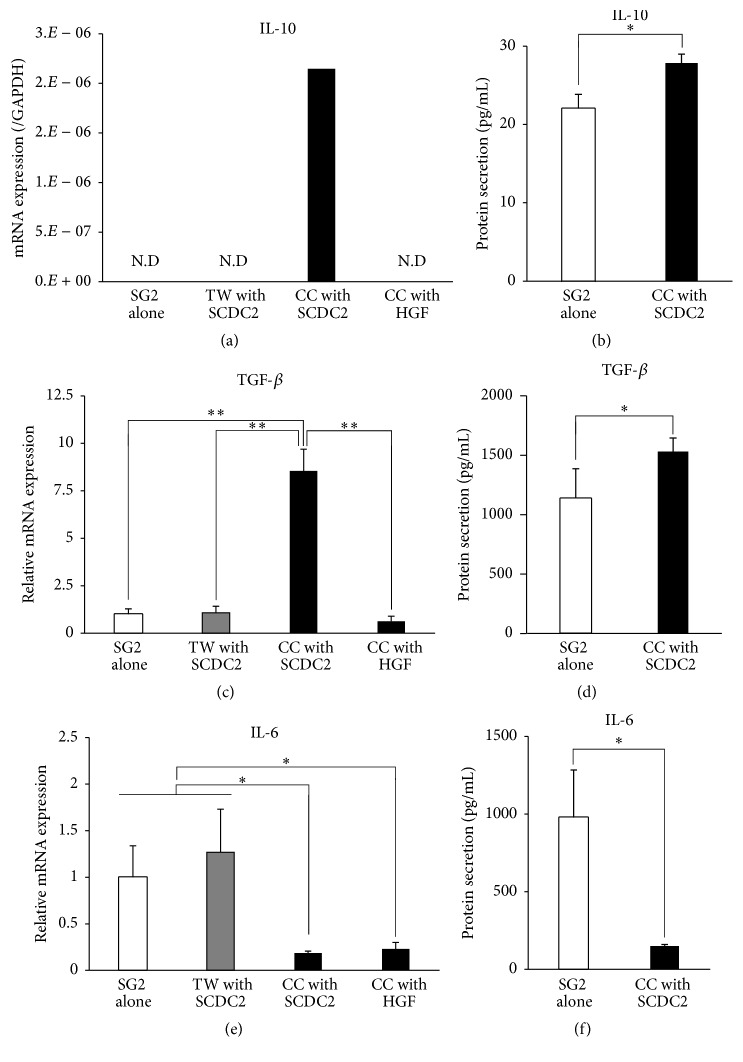
Expression levels of the anti-inflammatory cytokines IL-10 and TGF-*β* in SG2 cells increased by direct-contact coculture between SG2 and SCDC2 cells, whereas IL-6 expression decreased. SG2 cells were cocultured with SCDC2 cells or HGFs in a direct (CC) coculture system and an indirect transwell (TW) coculture system. (a, c, e) mRNA expression levels were investigated by RT-qPCR using rat-specific primers. Reported values are normalized to* Gapdh* expression. The results are expressed as the fold change relative to the respective control (SG2 alone). (b, d, f) Cytokines produced from the SG2 cells were quantified using sandwich ELISA with mouse-specific antibodies that show no cross-reactivity with those of rats. Quantified target proteins are presented as the mean ± standard deviations. ^*∗*^*P* < 0.05, ^*∗∗*^*P* < 0.01.

Because secretion of MCP-1, an MSC chemoattractant, from PDL-Fs was increased by the stimulation with inflammatory cytokines, we hypothesized that the MCP-1-migrated MSCs would home to the inflammatory PDL tissue and make direct contact with PDL-Fs. Under the CC coculture system between MSCs and PDL-Fs, the levels of cytokine production in SG2 cells were investigated by RT-qPCR and ELISA using mouse-specific primers and antibodies. The mRNA and protein expression levels of the anti-inflammatory cytokines IL-10 and TGF-*β* were significantly higher in the CC coculture with SCDC2 cells than those in SG2 cells cultured alone (Figures 3(a), 3(b), 3(c), and 3(d)). Interestingly, the increased expressions of these anti-inflammatory cytokines were not observed under CC coculture with HGFs. Such promotion of the expression of anti-inflammatory cytokines from SG2 cells was not observed in the TW coculture system. In contrast, under the CC coculture with SCDC2 cells, the mRNA and protein expression levels of the inflammatory cytokine IL-6 in SG2 cells were significantly lower than those in SG2 cells cultured alone (Figures 3(e) and 3(f)), which was not observed in the TW coculture. Suppression of IL-10 expression was also observed under the CC coculture with HGFs. These results indicated that the anti-inflammatory activity of MSCs is induced by cell-to-cell contact between MSCs and PDL-Fs. However, the expression of another anti-inflammatory cytokine, IL-4, was not detected in any culture condition (data not shown).

### 3.4. MSC Stemness of SG2 Cells Is Enhanced by Direct Coculture with SCDC2 Cells

**Figure 4:**
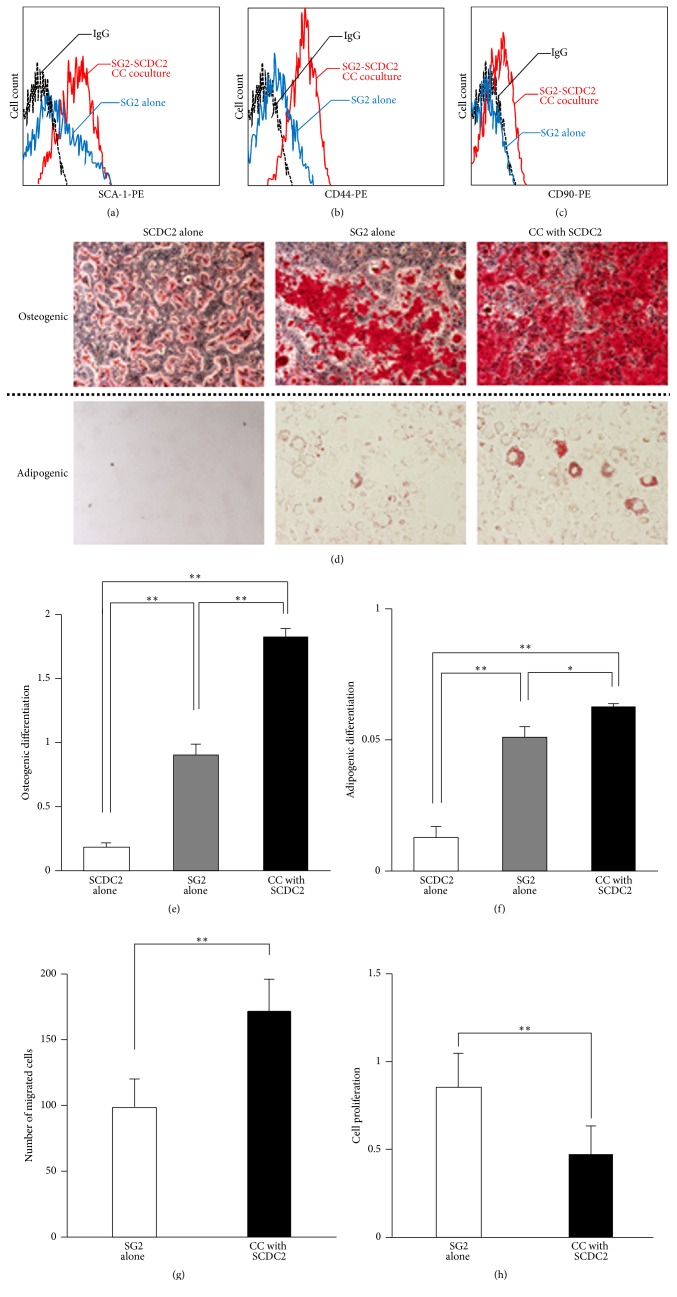
MSC stemness of SG2 cells is enhanced by direct coculture with SCDC2 cells. Cell-surface expression levels of SCA-1 (a), CD44 (b), and CD90 (c) were analyzed with each mouse-specific antibody in SG2 cells alone (blue), SG2 cells directly cocultured with SCDC2 cells (red), and an isotype control IgG (black) using flow cytometry. (d) SG2 cells were directly (CC) cocultured on the fixed feeder SCDC2 cells as described in Section 2. The SG2 cells were incubated in osteogenic (upper panel) or adipogenic (lower panel) induction medium. The cells were evaluated for extracellular matrix mineralization by alizarin red or lipid droplets by Oil Red staining. (e) Alizarin red was extracted with 10% cetylpyridinium chloride and absorbance was measured at 540 nm. (f) Oil Red O stain was extracted with DMSO and absorbance was measured at 540 nm. (g) The migratory ability of CC cocultured SG2 cells was investigated by a transwell migration assay. (h) The cell proliferation of CC cocultured SG2 cells was examined by a WST-1 assay. The results are expressed as the fold change relative to the respective control (SG2 alone). Data are presented as the mean ± standard deviation. ^*∗*^*P* < 0.05, ^*∗∗*^*P* < 0.01.

The cell-surface markers CD44, CD90, and SCA-1 on MSCs are known to independently and/or cooperatively play important roles for maintenance of the stemness of MSCs [45–47]. Therefore, we examined the expression of these MSC markers in SG2 cells that directly contacted SCDC2 cells. As shown in Figures 4(a)–4(c), the mouse MSC markers SCA-1, CD44, and CD90 were vigorously expressed in SG2 cells under the CC coculture system but not in SG2 cells cultured alone. In addition, the potential for osteogenic and adipogenic differentiation (Figures 4(d)–4(f)) as well as migration ability (Figure 4(g)) in SG2 cells was enhanced under the CC coculture system. Interestingly, coculture with SCDC2 cells decreased the proliferation of SG2 cells (Figure 4(h)). In general, cell proliferation is not compatible with differentiation and proliferation/differentiation switches in various cell types [48–50]. Therefore, these results suggest that the stemness of MSCs could be enhanced by their direct cell adhesion to PDL-Fs in damaged PDL tissues.

## 4. Discussion

Our previous study demonstrated that the expression of proinflammatory genes was promoted by stimulation with IL-1*β* and IL-6/sIL-6R in gingival fibroblasts [39]. In addition, expression of inflammation-related chemokines such as MCP-1 was enhanced by costimulation with IL-1*β* and IL-6/sIL-6R in gingival fibroblasts in the same study. MCP-1 is produced by many cell types, including fibroblasts and endothelial, epithelial, smooth muscle, mesangial, astrocytic, monocytic, and microglial cells [51–54]. Here, we focused on the effect of inflammatory cytokines on PDL-Fs, as another constituent cell type of the oral tissue. The results showed that the production of MCP-1 was significantly enhanced by stimulation with inflammatory cytokines such as IL-1*β*, IL-6/sIL-6R, and TNF-*α* in PDL-Fs and SCDC2 cells. MCP-1 transduces signals through the chemokine receptor CCR2, which is primarily involved in the recruitment of monocytes to the sites of inflammation, and is also expressed on MSCs [17, 55]. Previous studies have shown that MCP-1 induces the migration of human and murine BM-MSCs via transwell migration assays [56, 57] as well as in vivo experiments [58]. Some recent studies demonstrated that MCP-1 is one of the factors associated with the immune modulation caused by MSCs. In particular, MCP-1 secreted from MSCs induced the FasL-dependent apoptosis of T lymphocytes, and the apoptotic T cells then stimulated macrophages to secrete higher levels of TGF-*β*, which is associated with the generation of CD4^+^FoxP3^+^ regulatory T cells (Tregs) [59]. MCP-1 secreted from MSCs was also shown to exhibit an antiapoptotic effect on embryonic cardiomyoblasts through inhibition of caspase 3 [56]. Therefore, there is evidence to suggest the dual function of MCP-1 (proapoptotic or antiapoptotic), which might depend on the microenvironment surrounding the MSC-targeted cells. MCP-1 has been shown to induce MSCs to home towards various sites, including sites of inflammation, ischemic damage, trauma, or a developing malignant process, in an autocrine manner, and the cells then exhibit immunomodulating characteristics after homing [56]. Interestingly, in the present study, MCP-1 specifically induced the migration of BM-MSCs but not that of PDL-Fs, suggesting that MCP-1 specifically recruits MSCs to the inflammatory site but not normal fibroblasts in the damaged PDL tissue. To respond to this proposal, we investigated the mRNA expressions of CCR2 in SG2 and SCDC2 cells by RT-qPCR. Although expression levels cannot be compared because of different species and primers, mRNA of CCR2 was detected in both cells (data not shown). Therefore, the differential statuses of MCP-1-induced migratory activities between SG2 cells and SCDC2 cells may be due to the differential variances of intracellular signal transduction mechanisms downstream of CCR2 between these cells. We hypothesized that the migrated and homed BM-MSCs in the PDL tissue make active and direct contact with PDL-Fs in the PDL inflammatory tissue. MCP-1 is also involved in homing to inflamed tissues in immune-related cells such as macrophage and T cells [24]. On the other hand, MSCs suppress inflammation by immunosuppression against these cells [26, 27]. Therefore, chronicity of inflammation is suppressed by MSCs homed into inflamed tissues by MCP-1.

Many recent studies have demonstrated that MSCs exhibit their immunomodulatory properties by cell-to-cell contacts, as well as through the actions of secreted growth factors, cytokines, and chemokines [26, 27]. In this study, we demonstrated that the production of anti-inflammatory cytokines such as IL-10 and TGF-*β* increased in BM-MSCs by direct contact with PDL-Fs, whereas production of the inflammatory cytokine IL-6 decreased. We investigated the production of cytokines from SG2 cells cocultured with HGFs as the other cell types. As a result, mRNA expressions of IL-10 and TGF-*β* as anti-inflammatory cytokines from SG2 cells under coculture with HGFs were not enhanced. However, mRNA expression of IL-6 as an inflammatory cytokine was suppressed in HGFs as well as coculture with SCDC2 cells. These results strongly suggested that periodontal fibroblast SCDC2 cells specifically enhance the expression of anti-inflammatory cytokines in MSCs by the cell-to-cell adhesion between these cells. Therefore, MSCs that adhere to PDL-Fs play an immunomodulatory role that possibly facilitates the termination of inflammation during the wound healing process. One of the most prominent immunomodulatory cytokines produced and constitutively secreted by MSCs is TGF-*β*. As a pleiotropic cytokine, TGF-*β* regulates multiple fundamental cellular functions, including proliferation, differentiation, migration, adhesion, and apoptosis, that affect numerous biological processes such as development, wound healing, carcinogenesis, angiogenesis, and immune responses [60]. In addition, IL-10 is the cytokine most commonly discussed in relation to the immunoregulatory ability of MSCs. Although the conditions under which MSCs are most likely to secrete IL-10 are not definitely determined, it has been shown that MSCs secrete factors that upregulate the secretion of IL-10 by peripheral blood mononuclear cells [61], as well as by tolerogenic macrophages [62] and dendritic cells [63–65]. Yan et al. [66] reported that MSC-exposed Tregs have greater immunosuppressive capability than those that have not been cocultured with MSCs. They further suggested that IL-10 might be responsible for the enhanced suppressive capability of the MSC-exposed Treg cells. IL-6 has recently been demonstrated to be a pleiotropic cytokine, with a key role in a multitude of processes such as regulation of the immune response, hematopoiesis, inflammation, cell survival, apoptosis, cell proliferation, and oncogenesis [67, 68]. Cells expressing gp130 can bind to the IL-6/sIL-6R complex in a process known as transsignaling, which makes many cell populations susceptible to the effects of IL-6 [69, 70]. IL-6 secretion from MSCs has been demonstrated in both mice and humans [71, 72], and is detected either after induction with TNF*α*, IL-1*β*, and IFN*γ*, or spontaneously [63, 71, 73–76].

In addition, the expression levels of the mouse MSC markers SCA-1, CD44, and CD90 in BM-MSCs were upregulated by their cell-to-cell contact with PDL-Fs. As described above, various kinds of cell-surface markers, including CD44, CD90, and SCA-1, have been identified in mouse BM-MSCs [77]. CD44 is known to positively regulate the survival and migration of MSCs [45]. A previous report demonstrated that CD90 plays important roles in the attenuation of the commitment of MSCs, possibly resulting in the maintenance of their multipotency [46]. In addition, SCA-1 is known to positively regulate the self-renewal and survival of MSCs [47]. Thus, CD44, CD90, and SCA-1 independently and/or cooperatively play important roles for the maintenance of the stemness of MSCs. In fact, in the present study, the osteogenic and adipogenic differentiation status as well as migration ability of BM-MSCs directly cocultured with PDL-Fs were enhanced. Upon activation by tissue damage, MSCs contribute to the tissue repair process through a multitude of properties such as migration and differentiation. After homing into a damaged tissue, the cell-cell adhesion between MSCs and other types of cells is essential for MSC-dependent tissue regeneration in vivo. Our recent study suggested that the SCRG1/BST1 axis maintains the stemness and the expression of CD271, an MSC marker, of human BM-MSCs [25]. In addition, we demonstrated that the expression of another MSC marker, CD106, was dependent on cell density [78, 79]. Here, we demonstrated that the direct adhesion of MSCs to PDL-Fs enhances the stemness of MSCs, resulting in tissue repair and regeneration of the damaged PDL tissue.

Thus, we demonstrated that the anti-inflammatory and differentiation abilities of MSCs could be enhanced by their direct adhesion to PDL-Fs. Our findings provide a basis for the establishment of novel clinical strategies for periodontitis using MSCs.

## 5. Conclusions

Our results provide insight into the mechanism underlying the role of MSCs in tissue repair and regeneration of the PDL. In brief, MCP-1, which is secreted by fibroblasts under stimulation with inflammatory cytokines at a PDL inflammatory lesion, induces the migration and homing of BM-MSCs into the PDL inflammatory tissue. These MSCs that adhered to PDL-Fs play an immunomodulatory role to facilitate the termination of inflammation during the wound healing process. Furthermore, this adhesion between MSCs and PDL-Fs upregulates the expression of stem cell markers in MSCs, possibly resulting in the enhancement of their stemness to promote tissue repair and regeneration in the damaged PDL tissue.

## Figures and Tables

**Table 1:** Primer sequences for qRT-PCR.

Target gene	Reactivity	Primer (5′-3′)
*Gapdh*	Rat	F: GGCACAGTCAAGGCTGAGAATG
		R: ATGGTGGTGAAGACGCCAGTA
	Mouse	F: TGTGTCCGTCGTGGATCTGA
		R: TTGCTGTTGAAGTCGCAGGAG
*Mcp-1*	Rat	F: CTATGCAGGTCTCTGTCACGCTTC
		R: CAGCCGACTCATTGGGATCA
*Sdf-1*	Rat	F: GAGCCAACGTCAAACATCTGAA
		R: TCCAGGTACTCTTGGATCCACTTTA
*Il-4*	Mouse	F: ACGGAGATGGATGTGCCAAAC
		R: AGCACCTTGGAAGCCCTACAGA
*Il-6*	Mouse	F: CAACGATGATGCACTTGCAGA
		R: CTCCAGGTAGCTATGGTACTCCAGA
*Il-10*	Mouse	F: GCCAGAGCCACATGCTCCTA
		R: GATAAGGCTTGGCAACCCAAGTAA
*Tgf-β*	Mouse	F: TACGGCAGTGGCTGAACCAA
		R: CGGTTCATGTCATGGATGGTG
